# A Case Report of Neuromyelitis Optica Spectrum Disorder in a Young Patient With Systemic Lupus Erythematosus

**DOI:** 10.7759/cureus.83092

**Published:** 2025-04-27

**Authors:** Xaver Audhya, Jane Ma, Luciano Castaneda

**Affiliations:** 1 Medicine, Olive View University of California Los Angeles Medical Center, Sylmar, USA; 2 Hospital Medicine, University of California Los Angeles David Geffen School of Medicine, Los Angeles, USA

**Keywords:** anti-aquaporin 4(aqp4) antibody, area postrema syndrome, intractable nausea vomiting hiccups, neuromyelitis optica spectrum disorder (nmosd), sle with nmo

## Abstract

Neuromyelitis optica spectrum disorder (NMOSD), previously neuromyelitis optica, is an autoimmune, inflammatory CNS syndrome that can present in a variety of manifestations. It is associated with serum aquaporin-4 immunoglobulin G antibodies (AQP4-IgG); however, its presence is not required for the diagnosis. In this report, a rare case of NMOSD is presented in a patient with systemic lupus erythematosus (SLE).

The patient is a 21-year-old female with SLE who presented with intractable nausea, vomiting and hiccups. She underwent a thorough gastrointestinal workup that was non-revealing. A CNS workup was initiated ultimately revealing the diagnosis of NMOSD. Her symptoms resolved after initiating high-dose intravenous steroids and she was subsequently started on rituximab.

In patients presenting with area postrema syndrome, in the setting of an established autoimmune disorder, clinicians should have a high clinical suspicion of NMOSD. An MRI of the brain and checking for serum AQP-4 IgG are important in making the diagnosis.

## Introduction

Neuromyelitis optica spectrum disorder (NMOSD), formerly known as neuromyelitis optica (NMO), is a rare, autoimmune inflammatory CNS syndrome with a variety of symptoms that has been shown to be associated with serum aquaporin-4 immunoglobulin G antibodies (AQP4-IgG) [1-3]. In this report, we discuss a patient with systemic lupus erythematosus (SLE) who presented with area postrema syndrome that was ultimately diagnosed with NMOSD. Her NMOSD was thought to be a coexisting diagnosis from SLE.

## Case presentation

A 21-year-old female with a history of seizure disorder, systemic lupus erythematosus (SLE) with lupus nephritis, posterior reversible encephalopathy syndrome (PRES) and autoimmune hemolytic anemia (AIHA) presented with abdominal pain, nausea, and non-bloody, non-bilious emesis. Symptom onset was one week prior to presentation with her abdominal pain located in the lower quadrants and feeling like an ache which worsened after eating. No sick contacts, no recent travel, no new foods and no new medications were started. Her emesis started with solid foods and progressed to the point where she could not keep liquids down. She then developed an episode of diarrhea without hematochezia or melena. Dizziness started which was worse when getting up. She had two episodes when she fell to the ground after getting up too quickly, with associated body shaking. No post-ictal confusion or incontinence was noted. She was concerned about a possible seizure given her history, although her last seizure was as a child and she had since been taken off antiseizure medications.

Upon presentation to the emergency room, physical exam was notable for dry mucous membranes and no signs of acute abdomen. Pertinent labs on initial presentation are shown in Table 1. A CT Abdomen and Pelvis showed colonic wall thickening, otherwise no acute pathology was seen. She was given anti-emetics and failed a diet challenge; therefore, admission was requested.

**Table 1:** Laboratory results on early presentation WBC: white blood cell; Hgb: hemoglobin; AST: aspartate aminotransferase; ALT: alanine aminotransferase; LDH: lactate dehydrogenase; INR: international normalized ratio; ESR: erythrocyte sedimentation rate; CRP: C-reactive protein; C3: complement protein 3; C4: complement protein 4

Test	Result	Reference Range
WBC	2.5 K/cumm	4.5-10.0 K/cumm
Hgb	12.9 g/dL	12.0-14.6 g/dL
Platelets	265 K/cumm	160-360 K/cumm
Alkaline Phosphatase	44 U/L	38-126 U/L
AST	13 U/L	15-41 U/L
ALT	8 U/L	14-54 U/L
Total Bilirubin	1.3 mg/dL	0.4-1.2 mg/dL
Lipase	92 U/L	22-51 U/L
LDH	109 U/L	98-192 U/L
Haptoglobin	82 mg/dL	36-195 mg/dL
Reticulocyte Count	25x10(9)/L	29-116x10(9)/L
ESR	8 mm/hr	0-29 mm/hr
CRP	1.0 mg/L	0-7.0 mg/L
C3	68 mg/dL	90-180 mg/dL
C4	15.4 mg/dL	10-40 mg/dL

On admission, the patient further revealed that her presenting symptoms were similar to when she was diagnosed with SLE. Rheumatology was therefore consulted to evaluate for lupus involvement in the setting of the patient being non-adherent to her lupus regimen. Complement levels, as shown in Table 1, were non-revealing. At that time, an SLE flare was thought to be unlikely given her normal inflammatory markers.

AIHA was evaluated as she had an elevated total bilirubin with a decline in her hemoglobin on a repeat lab check. Additional studies, including lactate dehydrogenase (LDH), haptoglobin and her reticulocyte count, were not consistent with AIHA (Table 1).

Her symptoms persisted; therefore, Gastroenterology was consulted. In addition to her intractable nausea and vomiting, she endorsed development of intractable, severe hiccups. Given the absence of known organic etiologies for her symptoms, she underwent an esophagogastroduodenoscopy (EGD). Her EGD revealed a small hiatal hernia that did not explain her symptoms. An upper GI series was done, which was non-revealing. She was started on standing reglan and baclofen, which helped reduce her symptoms, but she continued to have episodes of emesis.

**Figure 1:**
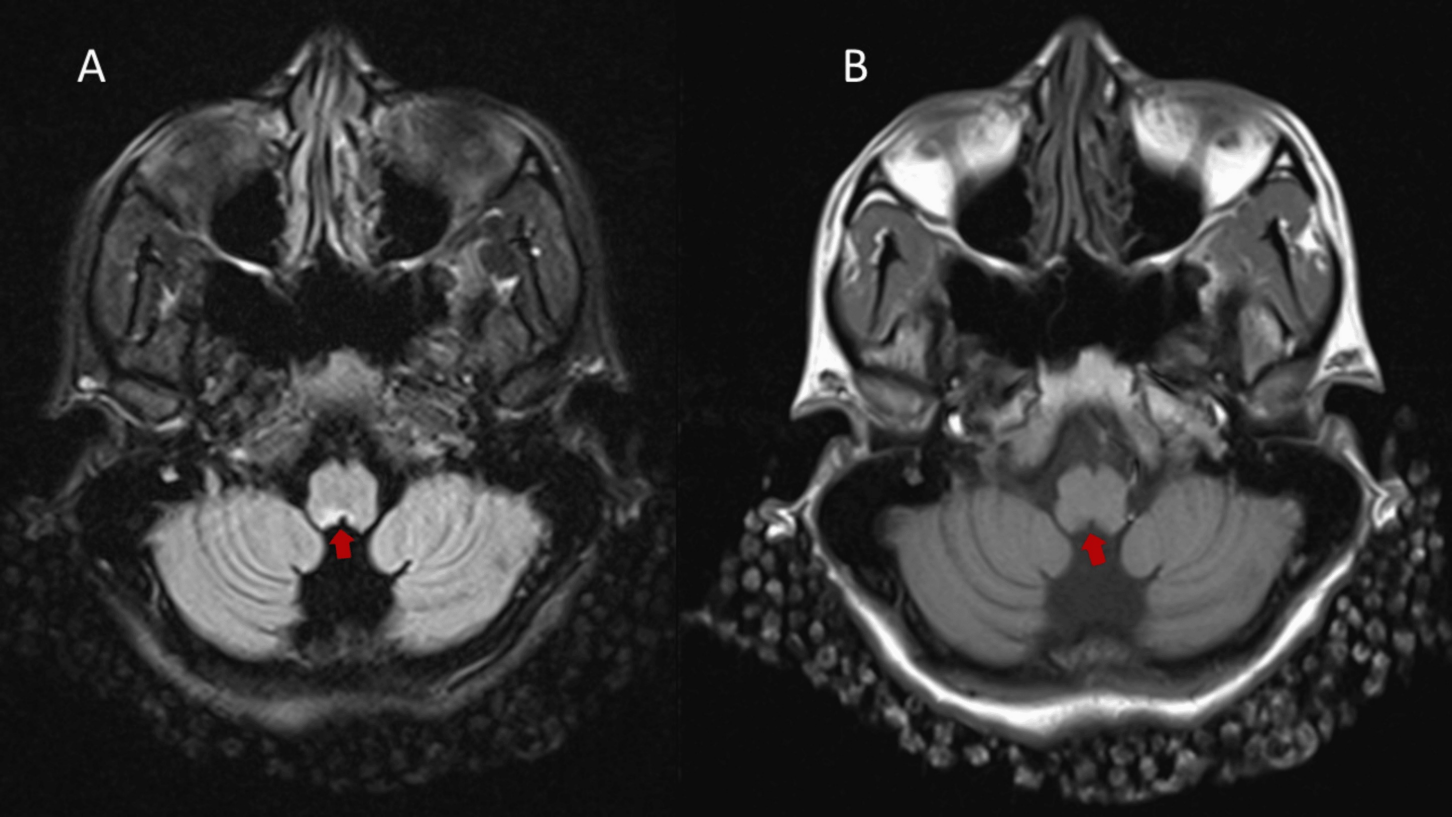
(A) T2/fluid-attenuated inversion recovery (FLAIR) hyperintensity at the right greater than left dorsal medulla. (B) T1 hypointensity at the right greater than left dorsal medulla. The arrows point to the lesion in the dorsal medulla, emphasizing the right.

Given her unremarkable GI workup, a trial of low-dose steroids was started per recommendation by Rheumatology with no improvement in symptoms. An MRI Brain was ordered to evaluate for central causes and demonstrated encephalomalacia at the dorsal medulla abutting the caudal recess of the fourth ventricle/obex bilaterally (Figure 1). This finding was concerning for old infarction vs NMOSD vs Bickerstaff encephalitis. Neurology was consulted and felt the etiology of her symptoms was secondary to NMOSD. A serum AQP4-IgG was sent. She met two of the diagnostic criteria in the absence of results for the AQP4-IgG, which included area postrema syndrome and MRI findings in the dorsal medulla lesion. She was started on IV solumedrol 1g daily for five days, after which her symptoms resolved by her second dose. An MRI Cervical and Thoracic spine were obtained resulting in areas of demyelination/myelitis in the C spine. A lumbar puncture was done and a multiple sclerosis (MS) panel was sent (Table 2). She was started on rituxan by discharge as B-cell inhibition was favored from an MS or NMO perspective. She was discharged with a prednisone taper and with plans to continue rituxan. Results for the NMO antibody AQP4 returned positive the day after discharge while the MS panel was pending by discharge.

**Table 2:** Pertinent CSF Results CSF: cerebrospinal fluid; RBC: red blood cell; IgG: immunoglobulin G

CSF Test	Result	Reference Range
RBC	1/cumm	0-5/cumm
Nucleated Cell Count	0/cumm	0-5/cumm
Glucose	72 mg/dL	40-70 mg/dL
Protein	23 mg/dL	15-45 mg/dL
Oligoclonal Bands	absent	absent
Myelin Basic Protein	<2.0 mcg/L	<2.0mcg/L
IgG	2.8 mg/dL	0.8-7.7 mg/dL
IgG Index	0.46	< 0.70

## Discussion

Neuromyelitis optica has expanded to the term neuromyelitis optica spectrum disorder as there have been numerous manifestations recognized in a range of diseases associated with the serum AQP4-IgG in up to 80% of those diagnosed with the disease [1,2]. It has also been associated with another antigenic target called myelin oligodendocyte glycoprotein (MOG) [4]. It is an autoimmune, inflammatory CNS syndrome that affects females more proportionally than males, typically in those between the ages of 30 to 40, similar to the patient discussed here [3].

NMOSD can be classified into six core clinical syndromes by location. These include the optic nerve, spinal cord, area postrema of the dorsal medulla, cerebrum, diencephalon, and other brain stem regions, with the first three being the most common [2]. Area postrema syndrome consists of intractable vomiting and hiccups. This is the presenting syndrome in 10% of patients, additionally occurring at any time in up to 15 to 40% of patients with NMOSD [5]. In up to two-thirds of patients, area postrema syndrome will appear prior to an optic neuritis or myelitis attack [5].

The diagnosis for NMOSD can be broken down into two categories: (1) whether the patient is positive for AQP4-IgG or (2) the patient is negative for AQP4-IgG or the status is unknown. If a patient is positive for AQP4-IgG, they need one or more of the six core clinical syndromes mentioned above with emphasis on making a distinction from MS or other disorders [1]. For those with a negative or unknown AQP4-IgG, criteria include two or more of the six core clinical syndromes with one being from the first three mentioned with specific MRI findings and no alternate diagnosis [1,2]. For acute optic neuritis, the MRI must show either nonspecific white matter lesions or no abnormalities [1]. For acute myelitis, the MRI must show an extensive cord lesion and for area postrema syndrome, it must show a dorsal medullary lesion [1].

The course of the disease may include a relapsing course in up to 90% of patients, with the time interval between attacks varying from months to years [2]. Similar to MS, relapses result in an increase in disability over time. If untreated, up to 40% of patients become blind in one eye and up to 25% have their gait affected, requiring assistance [6].

NMOSD is associated with an autoimmune disease in up to 33% of patients, with SLE being among the more common [2]. Often times neurologic complications in those with an established rheumatologic condition are attributed to the rheumatologic condition; however, the presence of AQP4-IgG indicates the co-existence of NMOSD, such as in this patient [7].

Treatment for acute onset and relapses includes intravenous glucocorticoids while immunotherapy is intended to reduce the probability of relapses. Immunotherapy is used for both seropositive and negative patients. The most common agents used are rituximab, mycophenolate and azathioprine with rituximab showing a benefit in seropositive patients [8]. Eculizumab, inebilizumab and satralizumab are the three approved monoclonal antibody therapies with no biomarkers involved in drug selection aside from the presence of APQ4-IgG [2]. Treatment is recommended indefinitely as high relapse rates have been shown when stopping immunotherapy [9]. Additionally, monitoring lesions on an MRI and monitoring APQ4-IgG titers is not recommended.

## Conclusions

For our patient, the diagnosis was made prior to the results of the AQP4-IgG status being known. She presented with area postrema syndrome with her MRI Brain showing a lesion at the dorsal medulla and no alternative diagnosis. Her results did return following discharge as being positive for APQ4-IgG and eventually showed her MS panel was unremarkable. There was initial consideration for her symptoms being secondary to SLE; however, her unremarkable inflammatory markers made this unlikely. Furthermore, her resulting seropositivity for AQP4-IgG confirmed the co-existence of SLE with NMOSD. She was started on high-dose steroids, resulting in resolution of her intractable emesis and hiccups, followed by rituximab to reduce her chance of relapse. NMOSD should be considered in a young patient with area postrema syndrome with a higher clinical suspicion if the patient has an established autoimmune rheumatologic disorder.
